# Homocysteine promotes atherosclerosis through macrophage pyroptosis via endoplasmic reticulum stress and calcium disorder

**DOI:** 10.1186/s10020-023-00656-z

**Published:** 2023-06-12

**Authors:** Shan Zhang, Ying Lv, Xing Luo, Xiuzhu Weng, Jinyu Qi, Xiaoxuan Bai, Chen Zhao, Ming Zeng, Xiaoyi Bao, Xinyu Dai, Ying Zhang, Yuwu Chen, Minghao Liu, Sining Hu, Ji Li, Haibo Jia

**Affiliations:** 1 Department of Cardiology, The 2nd affiliated Hospital of Harbin Medical University, Harbin, 150001 People’s Republic of China; 2 National Key Laboratory of Frigid Zone Cardiovascular Diseases (NKLFZCD), Harbin Medical University, Harbin, 150001 People’s Republic of China; 3 Department of Gynaecology and Obstetrics, The First Affiliated Hospital of Jiamusi University, Jiamusi, 154007 People’s Republic of China

**Keywords:** Atherosclerosis, Calcium disorder, Homocysteine, Hyperhomocysteinemia, Inflammasome, Pyroptosis, Risk factor

## Abstract

**Background:**

Elevated plasma homocysteine levels, known as hyperhomocysteinemia, have been identified as an independent risk factor for atherosclerosis and related cardiovascular diseases. Macrophage pyroptosis-mediated inflammation is crucial in the development of atherosclerosis, but the underlying mechanisms remain unclear.

**Methods:**

A hyperhomocysteinemia atherosclerotic model with ApoE^−/−^ mice fed with a high-methionine diet was constructed to investigate the role of plasma homocysteine in atherosclerosis. THP-1-derived macrophages were used to investigate the mechanisms by which Hcy regulates pyroptosis.

**Results:**

We found that hyperhomocysteinemia resulted in larger atherosclerotic plaques and more secretion of inflammatory cytokines, while these effects were attenuated in Caspase-1 knockdown mice. Likewise, in vitro experiments demonstrated that treatment of macrophages with homocysteine resulted in NLRP3 inflammasome activation and pyroptosis, as evidenced by cleavage of Caspase-1, production of downstream IL-1β, elevation of lactate dehydrogenase activity, and extensive propidium iodide-positive staining of cells. These were all inhibited by Caspase-1 inhibitor. In addition, excessive generation of reactive oxygen species was associated with mitochondrial dysfunction, characterized by loss of mitochondrial membrane potential and ATP synthesis. Moreover, further experiments revealed that homocysteine induced endoplasmic reticulum stress, enhanced communication between the endoplasmic reticulum and mitochondria, and consequently contributed to calcium disorder. Furthermore, the endoplasmic reticulum stress inhibitor, 4PBA, the calcium chelator, BAPTA, and calcium channel inhibitor, 2-APB significantly improved macrophage pyroptosis.

**Conclusion:**

Homocysteine accelerates atherosclerosis progression by enhancing macrophages pyroptosis via promoting endoplasmic reticulum stress, endoplasmic reticulum-mitochondria coupling, and disturbing of calcium disorder.

**Supplementary Information:**

The online version contains supplementary material available at 10.1186/s10020-023-00656-z.

## Introduction

Atherosclerosis (AS) is an inflammatory disease influenced by genetic and acquired risk factors (Wolf and Ley 2019). Progression of the disease results in arterial atherosclerotic plaques and leads to clinical complications, such as myocardial infarction (Libby 2021). It is characterized by lipid accumulation within the arterial wall that consequently leads to a chronic, maladaptive, non-resolving inflammatory process (Fredman and MacNamara 2021). This process is the leading cause of cardiovascular events. Despite substantial scientific advances over the past decades, the pathomechanism of AS still requires more detailed investigation. Hyperhomocysteinemia (HHcy) is a potent independent risk factor for AS (Paganelli et al. 2021). According to several studies, there is strong evidence that elevated plasma homocysteine (Hcy) levels are closely associated with increased cardiovascular mortality and long-term adverse events in patients with coronary artery diseases (Zhao et al. 2022), (Peng et al. 2015). Additionally, HHcy is believed to promote unstable plaque formation (Zhang et al. 2021b, a). Moreover, previous studies have reported that Hcy accelerates AS and inflammatory macrophage accumulation (Xiong et al. 2021); however, the underlying molecular mechanisms have not been fully elucidated.

Pyroptosis, a form of programmed cell death, is characterized by cell swelling, protrusion of large bubbles from the plasma membrane, and cell lysis, and widely occurs in the initiation, progression, and complications of AS (He et al. 2021). It’s initiated by NLRP3 inflammasome assembly, leading to the activation of Caspase-1 (cleaved Caspase-1). Then, cleaved Caspase-1 promotes N-terminal Gasdermin D (cleaved GSDMD) maturation, which forms pores on the plasma membrane and contributes to pyroptosis (Wang et al. 2017), (Liu et al. 2016). Increasing evidence suggests that pyroptosis plays a critical role in AS progression. However, current studies have indicated that multiple drugs, such as melatonin (Zhang et al. 2018) and estrogen (Meng et al. 2021), attenuate AS plaque formation by inhibiting pyroptosis in endothelial cells (ECs). Previous studies have mostly focused on ECs pyroptosis in atherosclerotic lesions. Currently, as a type of regulated necrosis that secretes pro-inflammatory factors, macrophage pyroptosis has been reported to account for a large part of cell death during AS progression (He et al. 2021). However, the effects and detailed mechanisms of macrophage pyroptosis in AS have not been fully defined.

In recent decades, there has been a growing appreciation for the mechanisms of NLRP3 inflammasome activation. Mounting evidence demonstrates that uncontrolled endoplasmic reticulum stress (ERS) responses can activate the inflammasome and promote AS progression (Zhang et al. 2021b, a). The ERS response has been shown to cause thioredoxin-interacting protein to dissociate from thioredoxin, thereby activating the NLRP3 inflammasome to regulate inflammatory reactions and cell death via Caspase-1-dependent processing of mature IL-1β (Oslowski et al. 2012). In addition, the endoplasmic reticulum (ER) is a critical organelle that stores most of a cell’s calcium, and growing evidence suggests that calcium homeostasis is involved in the pathogenesis of AS (Shrestha et al. 2014). Similarly, studies have revealed that calcium participates in the activation of the NLRP3 inflammasome (Xian et al. 2022). However, the role of ERS and calcium disorders in Hcy-promoted AS remains unclear.

In the present study, we used an atherosclerotic mouse model to investigate the role Hcy plays in regulating macrophage pyroptosis during the progression of atherosclerotic plaques. Further, we aimed to elucidate the underlying molecular mechanisms behind this process.

## Materials and methods

### Cell culture

Human monocytic THP-1 cells (ATCC, USA) were cultured in RPMI 1640 medium (Gibco, USA) containing 10% fetal bovine serum (Gibco, USA). Cell density never exceeded 1 × 10^6^ cells/mL. Culture medium was refreshed every 2 days, which was maintained at 37 °C, 95% air/5% CO_2_ humidified atmosphere. Human monocyte-derived macrophages were treated with 100 nM phorbol myristate acetate (PMA) for 24 h to obtain macrophages. After pretreatment with inhibitor VX765 (10 μM) or NAC (5 mM) or 4PBA (500 μM) or BAPTA (10 μM) for 1 h, the cells were treated with 10 μM Hcy for 24 h.

### Quantitative real-time PCR analysis

Total intracellular RNA was extracted and purified by Trizol reagent (Invitrogen, USA) according to manufacturer’s instructions. Reverse RNA transcription into cDNA using the Reverse Transcription kit (Roche, Switzerland) and the obtained cDNA was subjected to quantitative polymerase chain reaction (qPCR) using SYBR Green (Roche, Switzerland). The sequence of related genes was shown as follows:

NLRP3: forward: 5′-CCACAAGATCGTGAGAAAACCC-3′,reverse: 5′-CGGTCCTATGTGCTCGTCA-3′;

IL-1β: forward: 5′-ATGATGGCTTATTACAGTGGCAA-3′,reverse: 5′-GTCGGAGATTCGTAGCTGGA-3′;β-actin: forward: 5′-ACAACTTTGGTATCGTGGAAGG-3′,reverse: 5′-GCCATCACGCCACAGTTTC-3′.

### Western blot assay

Total protein was extracted from macrophages or mice blood vessels using RIPA lysis buffer supplemented with 1 mM PMSF (Beyotime, China). The protein concentration was measured by BCA Protein Assay Kit (Beyotime, China). Western blot gel was established using Epizyme PAGE Gel Fast Preparation Kit. The protein was separated by SDS polyacrylamide gel electrophoresis, and transferred onto PVDF membranes (Millipore, China). Subsequently, membranes were blocked with 5% skim milk for 1 h at room temperature, and then incubated with different antibodies at 4 °C overnight. After washing with TBST three times, membranes were incubated with secondary antibody for 1 h at room temperature, including antibodies against NLRP3, Caspase-1, cleaved Caspase-1, cleaved GSDMD, IL-1β (CST, USA, 1:1000), β-actin (Affinity, USA, 1:10,000) and so on. Western blot bands were examined and analyzed by Image Lab™ Software (Bio-Rad). The protein bands were treated with ECL working solution (Solarbio, China) and visualized using a gel imaging system (Tanon, China). The relative expression levels of the target proteins were determined with Image J software.

### Cell viability assay

Cell viability was detected using enhanced cell counting kit-8 (CCK-8) (Dojindo, Japanese). After treatment, it’s necessary to add 10 μL CCK8 working solution into each well of the 96-plate and incubated with the macrophages for 2 h at 37 °C. The absorbance at 450 nm was used as a reference standard to calculate the cell viability.

### JC-1 staining

Prepare JC-1 staining working solution according to the manufacturer's protocol and take 1 mL to cover cells, incubating at 37 ℃ for 15–20 min. And then wash cells twice with JC-1 buffer (1 ×), digest and centrifuge, resuspend cells with cell culture solution.

### PI/Hoechst double staining assay

THP-1-original macrophage cells were washed twice by cold PBS, and then stained by Hoechst33342 staining (5 μg/mL) and PI (2 μg/mL) at 4 °C for 30 min. Afterwards, cells were washed once by cold PBS and observed with a fluorescent microscope (DMI 4000B, Leica, Germany).

### LDH assay

The levels of lactate dehydrogenase (LDH) in cell supernatant were measured using the LDH assay kit (Nanjing Jiancheng Biology Engineering Institute, China) to evaluate cytotoxicity. Cell culture medium was collected and centrifuged at 1000 rpm for 10 min. Then, 20 µL supernatant was mixed with 25 µL substrate buffer and 5 µL coenzyme Ι, and the mixture was incubated at 37 °C for 15 min. Then, 25 µL DNPH was added into the mixture. After incubation at 37 °C for 15 min, 250 µL of 0.4 M NaOH solution was added into the mixture. The absorbance was measured at 450 nm using the microplate reader. The standard curve was established using pyruvic acid solution as the standard substance with concentrations range from 0 to 1 mM.

### Reactive oxygen species (ROS) assay

The total ROS was measured by fluorescence microscope using DCFH-DA (Beyotime, China). The cells seeded in a 24-well plate, were washed twice with PBS treatment and co-incubated with serum-free culture medium containing 10 μM DCFH-DA (30 min, 37 °C, in dark). The cells were washed thrice with PBS. The fluorescent intensities were measured using fluorescence microscope.

### Calcium detection

The levels of cellular calcium and mitochondrial calcium were measured by Fluo-4, AM (Beyotime, China) and Rhod-2, AM (Maokang, China) according to the manufacturer’s instructions, respectively. After treatment, the cells were washed with PBS for 3 times and incubated with working solution for 30 min in the cell incubator. The results were observed by the fluorescent microscope.

### Animal experiments and tissue harvest

The animal experimental procedures were approved by the institutional research ethics committee of the Second Affiliated Hospital of Harbin Medical University in accordance with the National Institutes of Health guide for the care and use of laboratory animals. 6–8 weeks male ApoE^‑/‑^ mice with a C57BL/6 background were purchased from Beijing Vital River Laboratory Animal Technology (Beijing, China) and were free of food and water and housed in the animal center of the Second Affiliated Hospital of Harbin Medical University (22 ± 2 °C, 55 ± 5% relative humidity with a 12 h light/dark cycle). These mice are randomly divided into three groups with different diets for 16 weeks: normal diet (ND, n = 10); high fat western‑type diet (21% fat, 0.15% cholesterol; MD12015; Medicine Ltd., Jiangsu, China) (HFD, n = 10); and high fat western‑type diet supplemented with 2% methionine (Zhang et al. 2020) (HM, n = 10). The body weight and blood glucose of mice were tested every two weeks. Additionally, the Caspase-1 knockdown mouse model was constructed and fed with high fat western‑type diet (Casp1^−/−^-HFD, n = 10); and high fat western‑type diet supplemented with 2% methionine (Casp1^−/−^-HM, n = 10), respectively. After 16 weeks, all mice were anesthetized with an intraperitoneal injection of sodium pentobarbital (50 mg/kg body weight) and then euthanized by cervical dislocation. The blood samples were harvested. The hearts and aortas were removed carefully and fixed with 4%paraformaldehyde (4 °C for 24 h) or kept in optimal cutting temperature compound for immunostaining.

### Biochemical analysis

Peripheral blood plasma samples were collected by centrifugation. The levels of total cholesterol (TC), triacylglycerol (TG), high-density cholesterol (HDL), and low-density cholesterol (LDL) were measured by commercial kits (Nanjing Jiancheng Biology Engineering Institute, China).

### Histopathological staining

The aortic roots were isolated from mice, embedded in optimum cutting temperature compound (OCT), and cut into 6 µm-thick sections. The aortic roots were detected by hematoxylin and eosin (HE) staining and Masson staining assay to evaluate the atherosclerotic lesions (Solarbio, China), and the lipid deposition was evaluated by Oil Red O staining kit (Nanjing Jiancheng Biology Engineering Institute, China).

### Immunofluorescence

Immunofluorescence staining was performed to detect the expression of NLRP3/Caspase-1/GSDMD in macrophages. Briefly, the histologic sections were fixed with cold acetone for 10 min, penetrated by 0.3% Triton X-100 for 15 min, and then blocked with goat serum. Subsequently, the sections were incubated with the antibody at 4 °C overnight, followed by incubation with Alexa Fluor-conjugated secondary antibody (Abcam, USA) in the dark for 1 h. The nuclei were stained by 4′,6-diamidino-2-phenylindole (DAPI; Beyotime, China) for 5 min. The sections were imaged under fluorescence microscope.

### Enzyme-linked immunosorbent assay (ELISA)

Plasma samples were collected from ApoE^−/−^ mice of different groups for 16 weeks. Plasma concentrations of IL-1β and Hcy in ApoE^−/−^ mice were determined by ELISA kit (Nanjing Jiancheng Biology Engineering Institute, China). The standard substance was dissolved in standard dilution and was subsequently diluted to 7 different gradient concentrations. The prepared samples and standards were added into the antibody-covered microplates and incubated in 37 ℃ incubator for 30 min. The plate was washed 5 times and added HRP Conjugate Reagent. After incubating for 30 min, the plate was washed 5 times. Then chromogen solution A, B were added for 10 min, and stop solution was used to terminate the reaction. The absorbance values were measured within 10 min by a microplate reader (Tecan infinite M200, Switzerland). The levels of IL-1β and Hcy were calculated with ELISAcalc software based on the standard curves.

### Transmission electron microscopy (TEM)

After treatment, the cells were collected by centrifugation and resuspended in 1 mL PBS. After centrifugation again, the supernatant was removed, and the samples were fixed with glutaraldehyde overnight at 4 ℃. Samples were postfixed in cacodylate-buffered 1% osmium tetroxide, dehydrated in ethanol series, and embedded in Poly/Bed 812. Cellular morphology was observed in different groups.

### Statistical analysis

All the results were presented as mean ± standard error of mean, and all the data were acquired from at least three independent experiments. Two-tailed Student’s t-test were used to compare data between two groups with normal distributed values. Mann–Whitney U test was used to compare data between two groups with non-normal distributed values. Statistical analysis was performed using SPSS 21.0 software. P < 0.05 was considered statistically significant.

## Results

### Hcy induces macrophage pyroptosis and promotes atherosclerotic lesions in ApoE^−/−^ mice

**Fig. 1:**
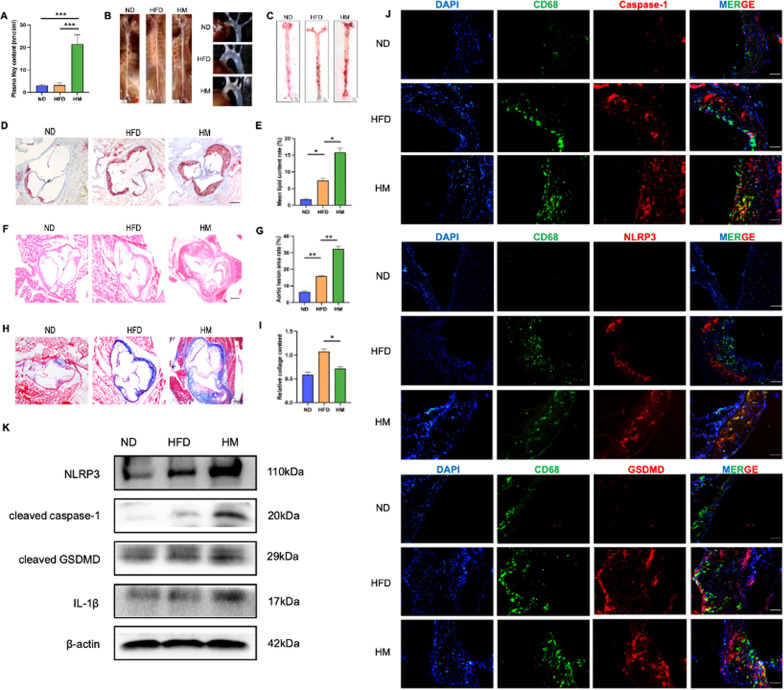
HHcy accelerates the progression of atherosclerosis in ApoE^−/−^ mice. **A** Plasma levels of Hcy assessed by ELISA. **B** The images of whole aorta and aortic arch. The scale bars correspond to 1 mm. **C** Representative images of Oil-red O staining of cardiac aorta in mice of two groups (n = 5). The scale bars correspond to 1 mm. **D** Oil-red O staining was performed on frozen aortic root sections from the two groups (n = 5). The scale bars correspond to 500 μm. **E** Relative quantitative analysis of lipid deposition. **F** HE staining was performed on the two groups (n = 5). The scale bars correspond to 500 μm. **G** Relative quantitative analysis of aortic root lesion areas. **H** Masson staining was performed to assess the relative collagen content (n = 5). The scale bars correspond to 500 μm. **I** The relative collagen contents were quantified. **J** Representative sections double stained for CD68 (green) and Caspase-1/NLRP3/GSDMD (red). The scale bars correspond to 100 μm. **K** Western blot result of pyroptosis-related proteins with aortas of two groups (n = 3). The data are shown as the mean ± SD. *P < 0.05, **P < 0.01, ***P < 0.001

To determine the successful establishment of the HHcy mouse model, plasma levels of Hcy were measured and showed a significant increase in the HM group (21 ± 5.45 nmol/ml), compared with the other two groups (3.5 ± 1.62 nmol/ml) (Fig. 1A). Images of the aortic arches were shown in Fig. 1B. Oil Red O staining revealed the presence of massive, red-stained lipids in both groups. The results indicated that methionine supplementation increased lipid deposition in the aortic roots and vessels (Fig. 1C–E). The areas of atherosclerotic lesions were significantly increased in the HM mice (Fig. 1F, G). The decreased collagen content was closely associated with unstable plaque formation. Masson staining indicated that the content of collagen was significantly decreased in the HM group compared to the ND and HFD group (Fig. 1H, I).

Moreover, we performed CD68/NLRP3, CD68/Caspase-1, and CD68/GSDMD double staining of histological sections of the aortic sinus from mice. The results showed that the expression levels of NLRP3, Caspase-1, and GSDMD in macrophages were remarkably increased in the mice exposed to methionine (Fig. 1J). Western blot also showed that NLRP3 inflammasome activation was increased in HM group (Fig. 1K). Taken together, these data indicate that Hcy could promote macrophage pyroptosis, thereby promoting plaque progression.

During the experiment, the baseline values of the mice were also monitored. After 16 weeks, mice in the HFD and HM groups showed similar weights, but were heavier than ND group (Additional file 1: Fig. S1A). The average daily food intake of each mouse was recorded to examine whether methionine influences appetite. The result showed that there was no significant difference in the daily intake of the ND, HFD, and HM mice (Additional file 1: Fig. S1B). Fasting blood glucose was recorded every 2 weeks, and no significant differences were observed between the mice of the HFD and HM groups, but higher than ND group (Additional file 1: Fig. S1C). Total cholesterol (TC), triglyceride (TG), LDL-C, and HDL-C levels were measured at the end of a 16-week feeding scheme. The results showed that there was no striking difference in serum TC, TG, LDL-C, and HDL-C levels between the HFD and HM groups, but changed compared with ND group (Additional file 1: Fig. S1D–G).

### Caspase-1 deletion alleviates AS and macrophage pyroptosis induced by Hcy

**Fig. 2:**
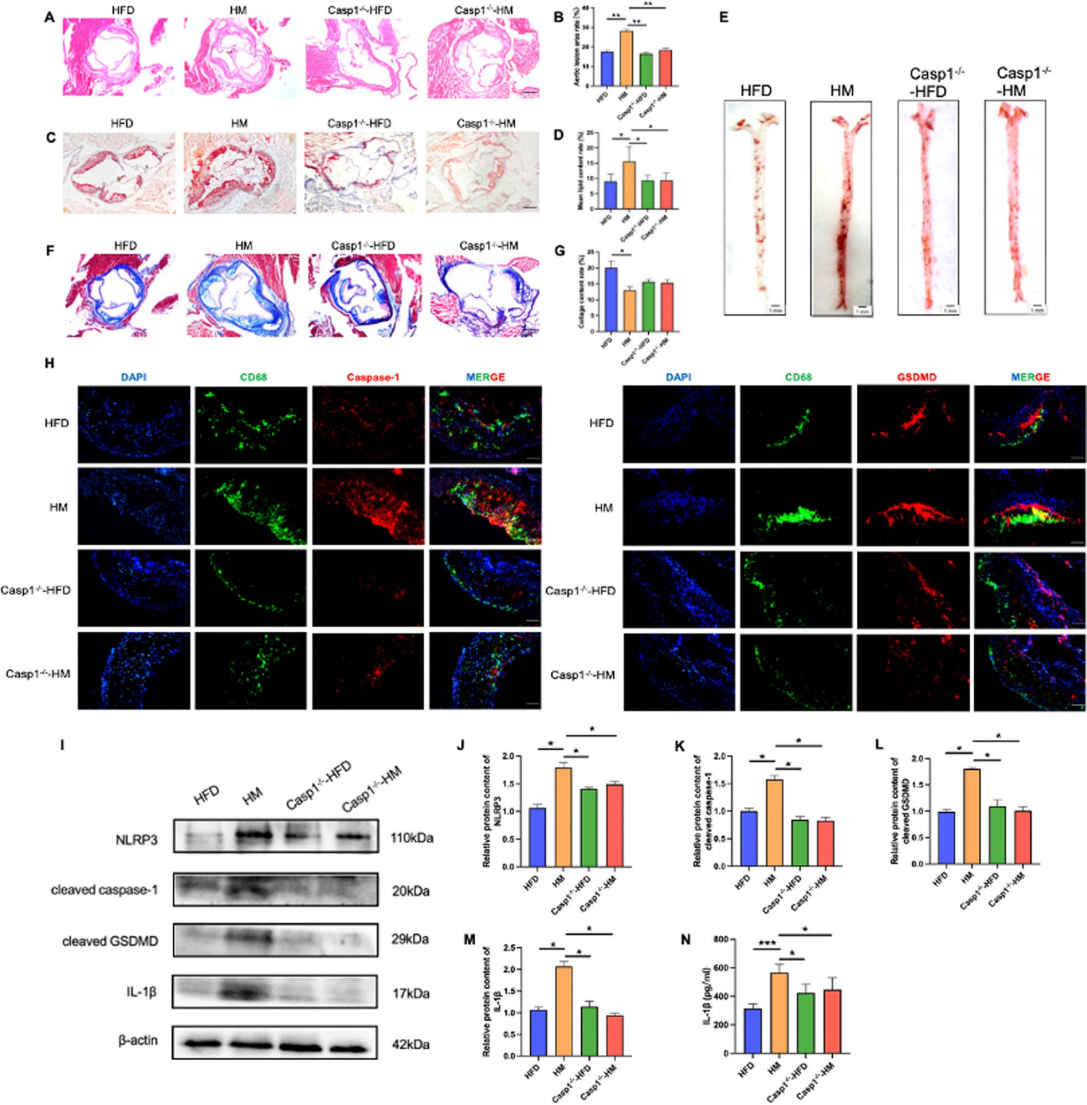
Mice were randomly divided into four groups with different diets for 16 weeks: HFD group (fed with high fat western-type diet), HM groups (fed with a methionine-supplemented diet), Casp1^−/−^ group (fed with a high fat diet), Casp1^−/−^ group (fed with a methionine-supplemented diet). **A** HE staining was performed on the four groups (n = 5). The scale bars correspond to 500 μm. **B** Relative quantitative analysis of aortic root lesion areas. **C** Oil-red O staining was performed on frozen aortic root sections from the four groups (n = 5). The scale bars correspond to 500 μm. **D** Relative quantitative analysis of lipid deposition. **E** Representative images of Oil-red O staining of cardiac aorta in mice of four groups (n = 5). The scale bars correspond to 1 mm. **F** Representative images of Masson staining (n=5). The scale bars correspond to 500 μm. **G** The relative collagen contents were quantified. **H** Representative sections double stained for CD68 (green) and Caspase-1/GSDMD (red) (n = 5). The scale bars correspond to 100 μm. **I** Western blot result of pyroptosis-related proteins with aortas of four groups (n = 3). **J**–**M** Relative protein expressions quantification of pyroptosis-related proteins. **N** Plasma levels of IL-1β assessed by ELISA (n = 10). The data are shown as the mean ± SD. *P < 0.05, **P < 0.01, ***P < 0.001

In order to further confirm that HHcy promotes AS by inducing macrophage pyroptosis, a Caspase-1 knockdown (Casp1^−/−^) mouse model was established, and the mice were divided into two groups, fed with a high fat diet or methionine-supplemented diet for 16 weeks. The above experiments were validated in Casp1^−/−^ mice. As mentioned, HE staining demonstrated that larger atherosclerotic plaques were observed in HM mice but were reduced in the Casp1^−/−^ group (Fig. 2A, B). Compared to HM group, the lipid content in the aortic sinus was also decreased in Casp1^−/−^ group. (Fig. 2C–E). Furthermore, in the HM group, Masson staining showed that Casp1 deletion significantly increased the content of collagen fibers in the aortic sinus in HHcy mouse model (Fig. 2F, G). These data suggested that Casp1 deletion decreased plaque area and lipid content in HM group, and increased plaque stability. The opposite results were observed in the Casp1^−/−^ group Immunofluorescence staining showed that the expression of Caspase-1 and GSDMD in the macrophages of the Casp1^−/−^ group was lower than that of the HM group (Fig. 2H). Western blot further showed that NLRP3 inflammasome activation was increased in methionine-treated mice but decreased in Casp1^−/−^ mice (Fig. 2I). Consistently, similar changes in the plasma concentrations of IL-1β were observed in methionine-treated mice (Fig. 2J).

### Hcy promotes NLRP3 inflammasome activation and pyroptosis in THP-1 macrophages

**Fig. 3:**
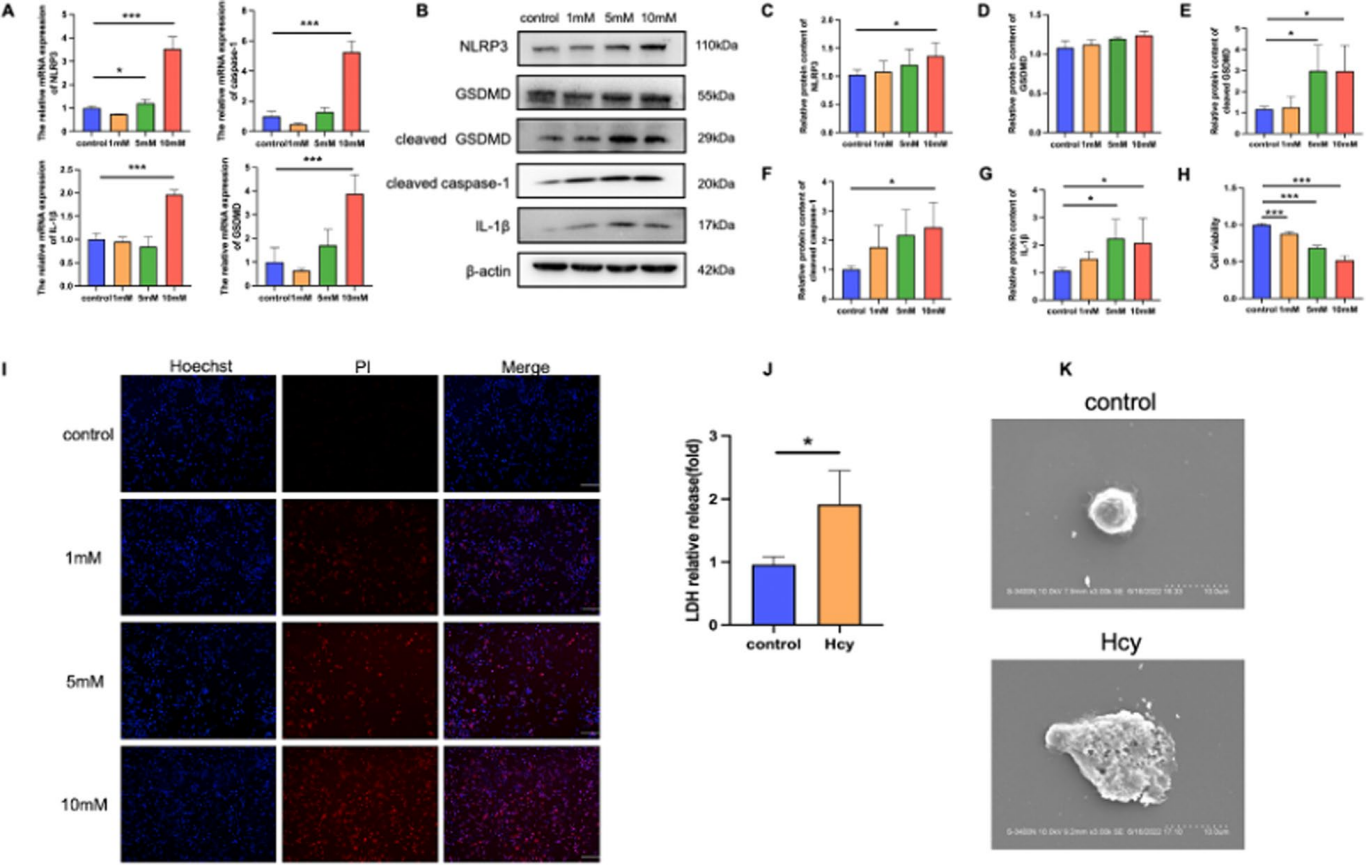
Hcy treatment triggers pyroptosis in THP-1. **A** Increases in the expression of pyroptosis-related genes (NLRP3 and IL-1β) at mRNA levels by different concentrations Hcy of 0, 1, 5, and 10 mM. **B** Western blot was used to evaluate the pyroptosis-related proteins level in different groups. **C**–**G** Relative protein expressions quantification of pyroptosis-related proteins. **H** Cell viability detected by CCK8. **I** The cell death was measured with Hoechst 33342 (blue)/PI (red) double-fluorescent staining. The scale bars correspond to 100 μm. **J** The levels of LDH were measured by commercial kits. **K** The morphology of cells was observed with scanning electron microscopy (SEM). The scale bars correspond to 10 μm. The data are shown as the mean ± SD. *P < 0.05, **P < 0.01, ***P < 0.001

THP-1 macrophages were used to investigate the mechanisms by which Hcy promoted NLRP3 inflammasome activation and pyroptosis. After the cells were treated with 1, 5, and 10 mM Hcy, the results revealed that the expression of NLRP3, IL-1β, and cleaved GSDMD was significantly increased (Fig. 3A–G). The CCK-8 was used to further characterize the Hcy-induced pyroptosis of macrophages, and the results showed that Hcy suppressed macrophage viability (Fig. 3H). During pyroptosis, pores can form in the cell membrane, leading to the release of cellular contents and the positive staining of dead cells, which can be determined using propidium iodide (PI) staining and lactate dehydrogenase (LDH) release assays, respectively. Our results showed Hcy-induced pore formation and membrane rupture, as indicated by the extensive PI-positive staining of cells (Fig. 3I) and increased LDH activity (Fig. 3J). Afterwards, 10 mM Hcy was chosen as the working concentration for the present study. In addition, scanning electron microscopy was used to observe the morphology of cells undergoing cell death, which showed cell swelling, large bubbles, and cell lysis after Hcy treated (Fig. 3K).

**Fig. 4:**
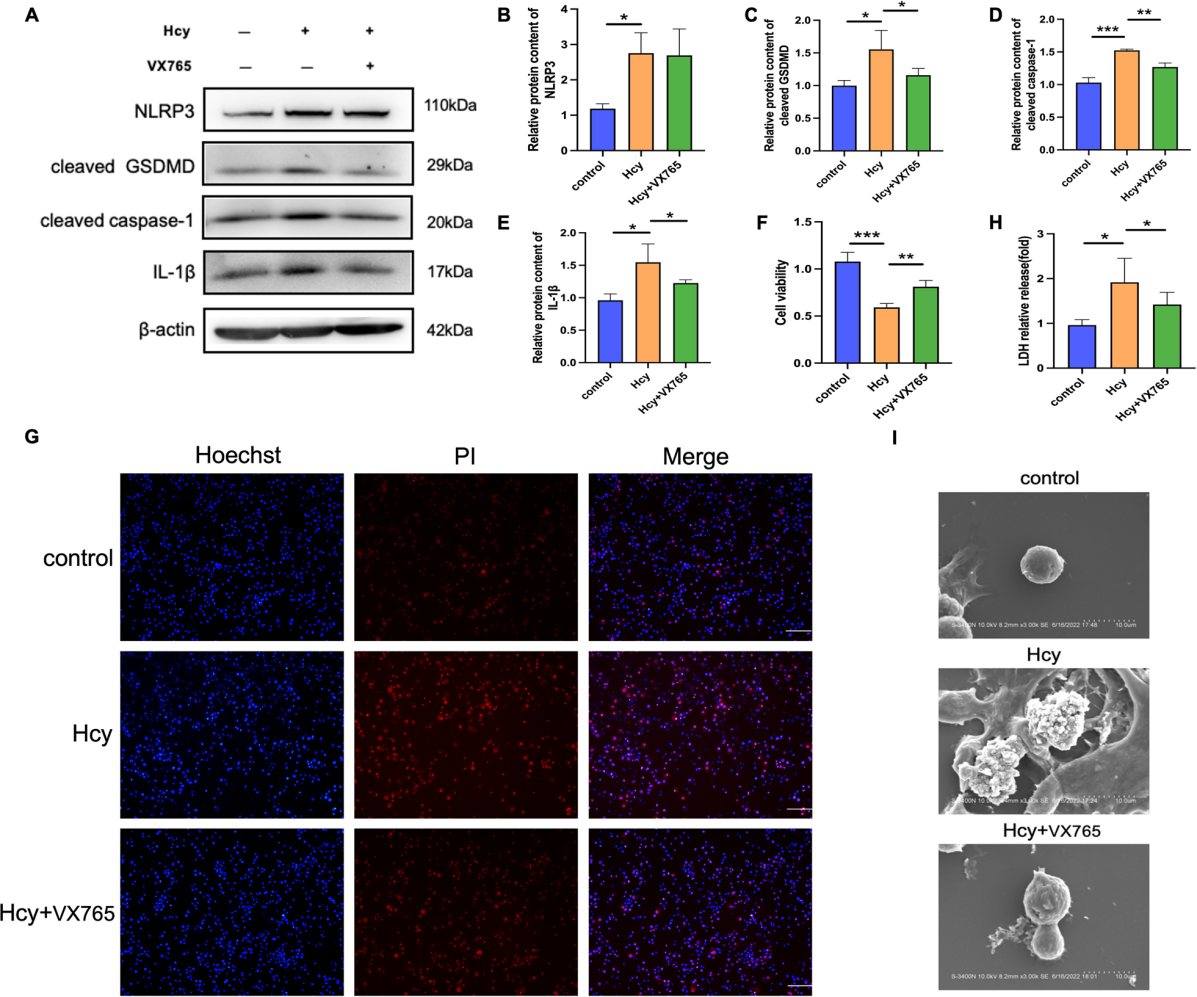
Caspase-1 inhibitor represses Hcy-induced macrophage pyroptosis. Macrophages were pretreated with caspase-1 inhibitor (VX-765, 10 μM) for 1 h, and then the cells were incubated with Hcy (10 mM) for 24 h. **A** Western blot was used to evaluate the pyroptosis-related proteins level in different groups. **B**–**E** Quantitative analysis of pyroptosis-associated protein expression. **F** Cell viability detected by CCK8. **G** The cell death was measured with Hoechst 33,342 (blue)/PI (red) double-fluorescent staining. The scale bars correspond to 100 μm. **H** LDH assay was used to evaluate the cell membrane integrity. **I** The morphology of cells was observed with scanning electron microscopy (SEM). The scale bars correspond to 10 μm. The data are shown as the mean ± SD. *P < 0.05, **P < 0.01, ***P < 0.001

To further investigate whether Hcy-induced pyroptosis of macrophages was Caspase-1-dependent, cells were pretreated with a Caspase-1 inhibitor. Our results showed that a Caspase-1 selective inhibitor (VX-765) decreased the level of activated Caspase-1 and inhibited the maturation of cleaved GSDMD and IL-1β (Fig. 4A–E). Cell lysis and pyroptotic cell death were reversed using VX-765, as demonstrated by the increase in cell viability (Fig. 4F) and percentage of PI-positive cells (Fig. 4G). Likewise, the increased LDH activity (Fig. 4H) and cell morphology (Fig. 4I) were partly reversed after VX-765 pretreatment. Overall, these results indicate that Hcy promotes macrophage Caspase-1-dependent pyroptosis.

### Hcy promotes macrophage pyroptosis by enhancing oxidative stress and mitochondrial dysfunction

**Fig. 5:**
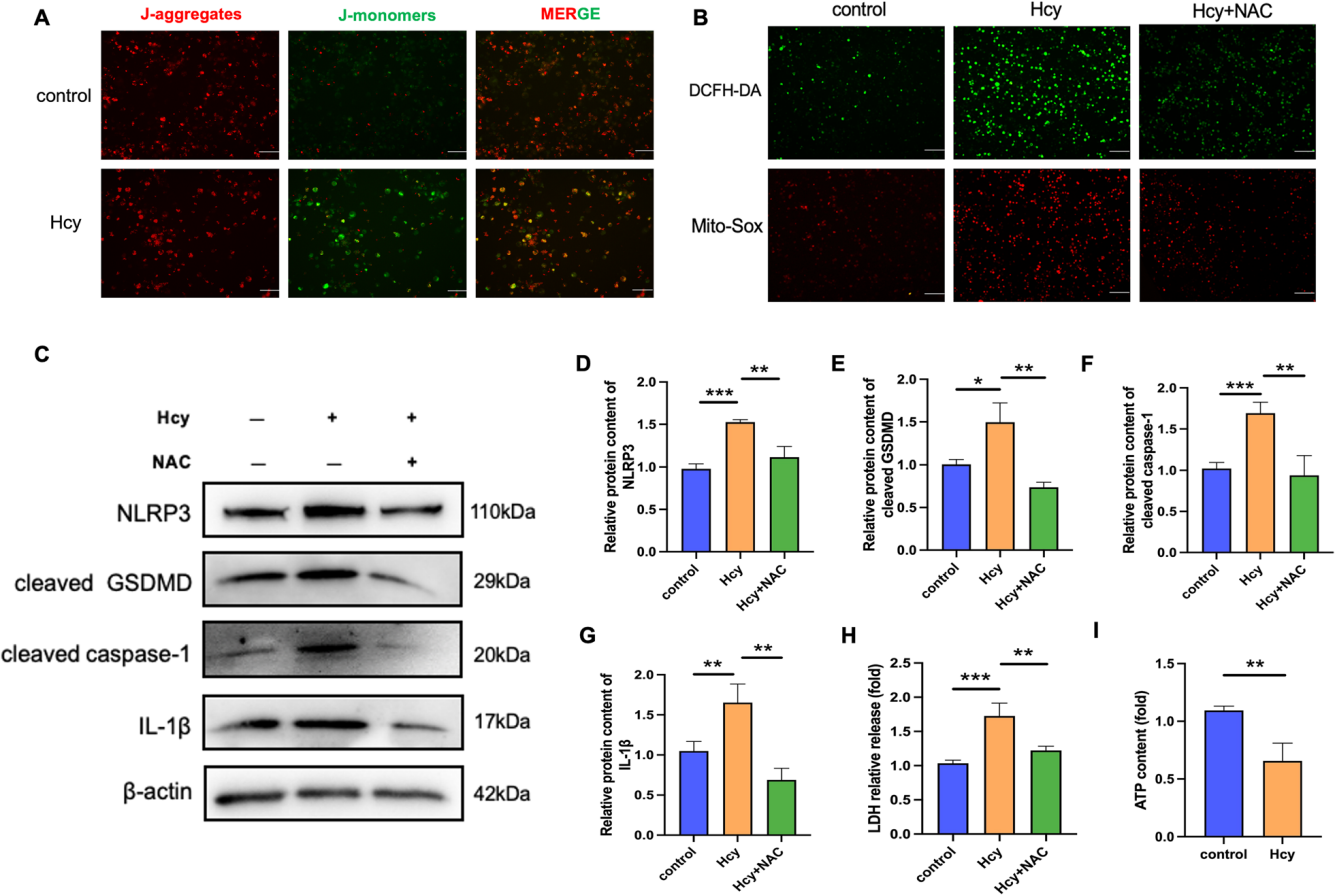
Pretreatment with ROS inhibitor suppresses Hcy-induced macrophage pyroptosis. Macrophages were pretreated with ROS inhibitor (NAC, 5 mM) for 1 h, and then the cells were incubated with Hcy (10 mM) for 24 h. **A** JC-1 assay was used to examine the mitochondrial membrane potential. The scale bars correspond to 100 μm. **B** Intracellular ROS level was detected using a DCFH-DA probe (green). Mitochondrial ROS level was detected by Mito-Sox Staining (red). The scale bars correspond to 100 μm. **C** Western blot was used to evaluate the pyroptosis-related proteins level in different groups. NAC pretreatment suppressed the upregulation of pyroptosis-associated protein levels in the presence of Hcy. **D**–**G** Quantitative analysis of pyroptosis-associated protein expression. **H** LDH assay was used to evaluate the cell membrane integrity. **I** The levels of ATP were measured by commercial kits after Hcy treated. The data are shown as the mean ± SD. *P < 0.05, **P < 0.01, ***P < 0.001

Given that reactive oxygen species (ROS) was essential for inflammasome activation, we investigated the effects of Hcy on mitochondrial function in macrophages by measuring mitochondrial membrane potential (MMP), total ROS, and mitochondrial ROS. Our results demonstrated that Hcy treatment dramatically decreased the MMP (Fig. 5A) and increased ROS levels in the macrophages. The treatment of macrophages with Hcy promoted intracellular ROS production and was suppressed by N-acetyl cysteine (NAC), a ROS inhibitor (Fig. 5B). NAC also inhibited the Hcy-induced protein expression of inflammasome components, including NLRP3, cleaved Caspase-1, IL-1β, and cleaved GSDMD (Fig. 5C–G), suggesting that NLRP3 inflammasome activation was dependent on ROS generation. Furthermore, NAC pretreatment reduced LDH activity in Hcy-treated macrophages (Fig. 5H). Mitochondria are the primary producers of adenosine 5′-triphosphate (ATP) for cell repair. Healthy mitochondria with a normal MMP and ultrastructure are the foundation of cellular ATP production. Conversely, when facing mitochondrial dysfunction, ATP generation was reduced (Fig. 5I). Taken together, these results indicate that Hcy promotes macrophage pyroptosis via ROS production.

### Hcy induces the endoplasmic reticulum stress response of macrophages

**Fig. 6:**
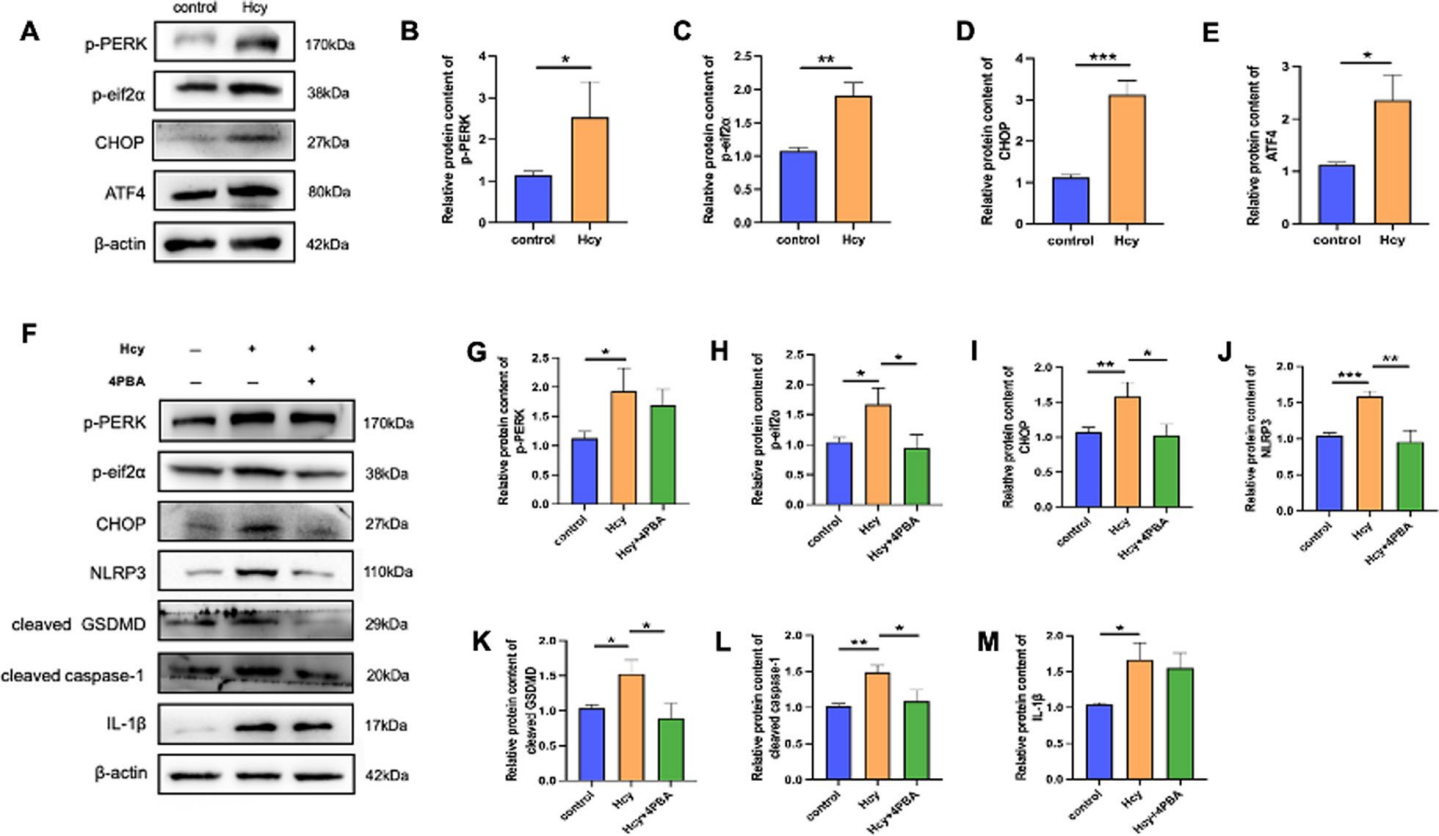
Hcy induced ER stress response of macrophages. **A** Western blot was used to evaluate the ERS-related proteins (p-PERK, p-eif2α, ATF4, CHOP) level after Hcy treated. **B**–**E** Relative protein expressions quantification of ERS-related proteins. **F** Western blot was used to evaluate the pyroptosis-related proteins level after ERS inhibitor 4PBA pretreatment. **G**–**M** Relative protein expressions quantification of ERS-related and pyroptosis-associated proteins. The data are shown as the mean ± SD. *P < 0.05, **P < 0.01, ***P < 0.001

Recent studies have suggested that ROS overproduction is correlated with mitochondrial dysfunction and ERS. To confirm whether ERS participated in macrophage pyroptosis induced by Hcy, we investigated the expression of ERS-related proteins. The expression of protein kinase R-like ER kinase (PERK), p-PERK, p-eIf2a, ATF4, and CHOP significantly increased in the Hcy-treated group (Fig. 6A–E). To further identify the underlying mechanisms of ERS in macrophage pyroptosis induced by Hcy, cells were pretreated with 4-PBA, a specific ERS inhibitor. Compared to the Hcy administration group, expression of the pyroptosis-related proteins, NLRP3, cleaved Caspase-1, cleaved GSDMD, and IL-1β, was significantly reduced after 4-PBA treatment (Fig. 6F–M), indicating that ERS plays a key role in Hcy-induced macrophage pyroptosis.

### Hcy promotes ER-mitochondria contacts closer in macrophages

Mitochondria-associated ER membranes (MAMs), structures connecting the ER and mitochondria, participate in energy metabolism, Ca^2+^ signaling, and mitochondrial dynamics. Moreover, Ca^2+^ signaling is critical for NLRP3 inflammasome activation. Thus, we further elucidated the potential mechanisms of Hcy in MAM.

**Fig. 7:**
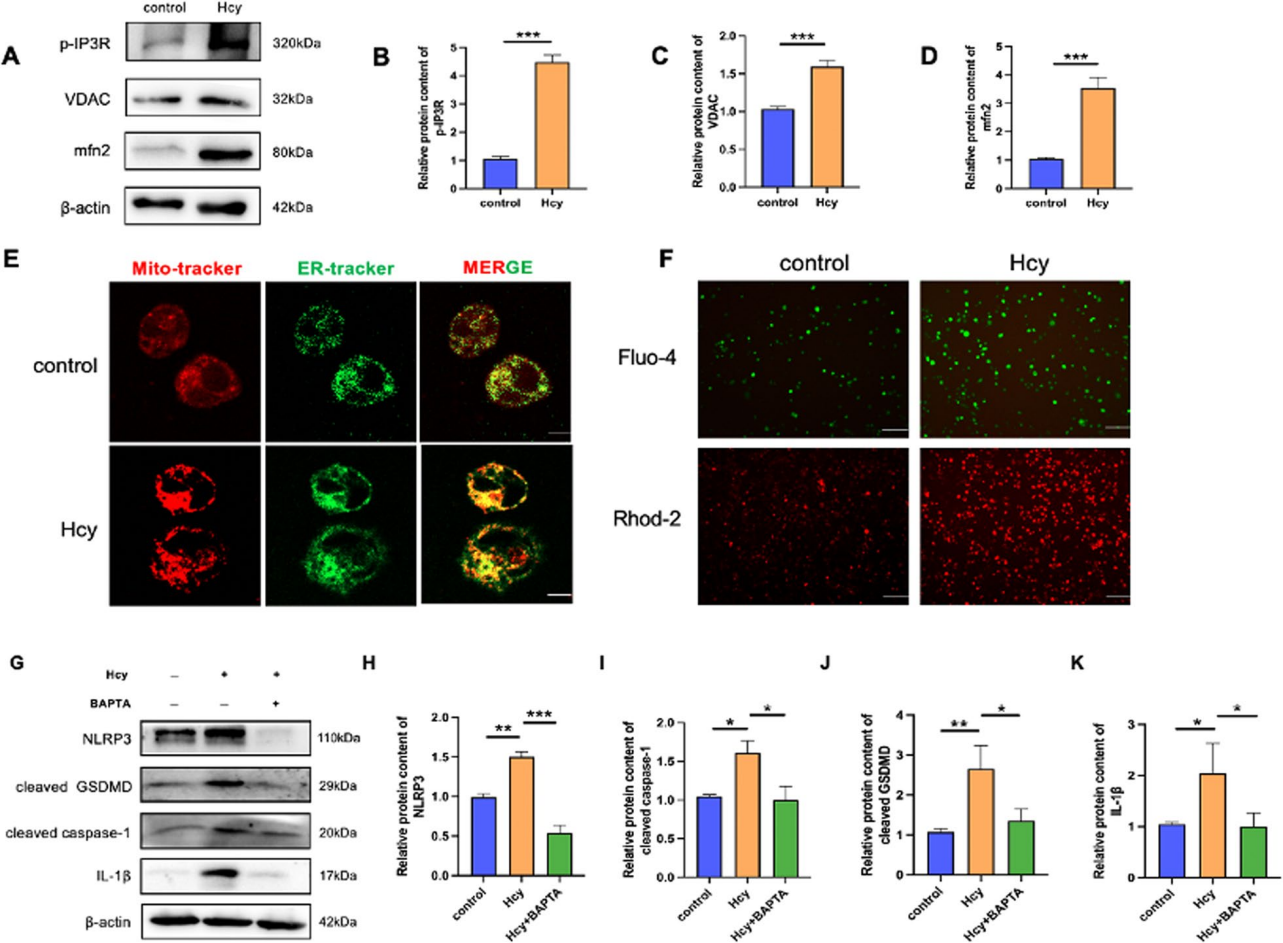
Hcy increases ER-mitochondria colocalization. **A** Western blot was used to evaluate the MAM-related proteins (p-IP3R, VDAC, mfn-2) level after Hcy treated. **B**–**D** Relative protein expressions quantification of MAM-related proteins. **E** The ER was stained with ER-tracker (shown in green), and mitochondria were stained with mito-tracker (shown in red) and then imaged using confocal microscopy. Colocalization was shown in yellow in the merged images. The scale bars correspond to 30 μm. **F** Ca^2+^ change with the fluorescence. The levels of cellular and mitochondrial calcium were measured by Fluo-4, AM and Rhod-2, AM, respectively. The scale bars correspond to 100 μm. **G** Western blot was used to evaluate the pyroptosis-related protein level after calcium chelator BAPTA pretreatment. **H**–**K** Relative protein expressions quantification of pyroptosis-associated proteins. The data are shown as the mean ± SD. *P < 0.05, **P < 0.01, ***P < 0.001

Voltage-dependent anion channel (VDAC), a mitochondrial protein, interacts with inositol-3-phosphate receptor (IP3R), an ER protein in MAMs, and controls ER/mitochondrial Ca^2+^ exchange. We found that the expression of p-IP3R, VDAC, and mitofusin 2 increased, indicating that Hcy could cause MAM disorder (Fig. 7A–D). Since this suggested that there were alterations made in ER-mitochondria tethering, we evaluated whether a similar increase would occur in response to Hcy by monitoring the colocalization of mitochondria and ER using Mito-tracker (Red) and ER-tracker (green) probes via confocal microscopy (Fig. 7E). The width of MAM influences Ca^2+^ transfer through the IP3R-VDAC complex, which assembles when the distance between both organelles is in the range of 10–25 nm. The close interaction between the mitochondria and ER facilitates Ca^2+^ crosstalk, and in turn regulates mitochondrial function. Therefore, to determine whether Hcy interfered with Ca^2+^ homeostasis in macrophages, Fluo-4 AM and Rhod-2 AM were used to detect the levels of cellular and mitochondrial Ca^2+^_,_ respectively. The results showed that Ca^2+^ accumulated in the cytosol and the mitochondrial matrix (Fig. 7F). To assess the relationship between Ca^2+^ and the NLRP3 inflammasome, cells were pretreated with BAPTA to bind Ca^2+^. After the Ca^2+^ concentration was buffered, pyroptosis-related proteins were consistently suppressed (Fig. 7G–K). To further confirm that Hcy causes pyroptosis by promoting the release of Ca^2+^ from endoplasmic reticulum, macrophages were pretreated with IP3R inhibitor 2-APB to block Ca^2+^ channels. Macrophage pyroptosis was reversed using 2-APB through PI-positive cells (Additional file 1: Fig. S2A) and cell morphology (Additional file 1: Fig. S2B). Likewise, cell viability (Additional file 1: Fig. S2C) and LDH activity (Additional file 1: Fig. S2D) were partly reversed after 2-APB pretreatment. The Fluo-4 and Rhod-2 results showed that Ca^2+^ accumulated in the cytosol and the mitochondrial matrix but decreased after 2-APB treatment (Additional file 1: Fig. S2E). When Ca^2+^ outflow from the endoplasmic reticulum was inhibited, pyroptosis-related proteins were consistently suppressed (Additional file 1: Fig. S2F–J). These results suggested that IP3R-mediated release of Ca^2+^ from endoplasmic reticulum may be the mechanism of Ca^2+^ homeostasis and pyroptosis induced by Hcy. In conclusion, MAM and Ca^2+^ disorders are essential for Hcy-induced macrophage pyroptosis.

## Discussion

Our data support that HHcy promotes atherosclerotic plaque progression in ApoE^−/−^ mice, partially by promoting macrophage pyroptosis. This study also provides promising evidence that Hcy promotes AS by inducing macrophage pyroptosis via ERS and calcium disorder. These results shed new insight into the mechanisms of Hcy-induced AS, advance our understanding of the pathophysiology of HHcy, and suggest potential therapeutic targets. In THP-1-derived macrophages, Hcy promoted ERS and ER-mitochondrial contact, which was the site of direct Ca^2+^ transfer between the ER and mitochondria. Overactivation of the electron transport chain in the presence of excessive mitochondrial Ca^2+^ is directly linked to enhanced cellular and mitochondrial ROS production, and consequently promotes NLRP3 inflammasome-mediated pyroptosis.

Recently, the mechanisms by which Hcy promotes AS have seen an increase in interest. It has been reported that HHcy accelerates AS by inducing endothelial dysfunction, smooth muscle cell proliferation (Bennett et al. 2016), and macrophage apoptosis (Cong et al. 2017) in vivo and in vitro. Nevertheless, the underlying mechanisms by which Hcy promotes AS require further investigation. Pyroptosis is a recently discovered form of inflammatory cell death mediated by the NLRP3 inflammasome and is dependent on Caspase-1 activation. Autopsy studies confirmed that the expression of NLRP3 and Caspase-1, the major components of the NLRP3 inflammasome, was significantly higher in atherosclerotic plaques and correlated with the markers of plaque instability (Naora et al. 1989). However, most studies have focused on endothelial cell pyroptosis in AS, but there is little evidence on the effects and mechanisms of macrophage pyroptosis. Our recent study has demonstrated that targeting macrophage pyroptosis contributes to the prevention of AS (Luo et al. 2022). However, there is still a lack of evidence regarding the role and mechanism of macrophage pyroptosis in Hcy-induced AS. In the present study, our data revealed that Hcy promoted the activation of the NLRP3 inflammasome and macrophage pyroptosis, secondary to inflammation, all of which were thought to contribute to atherogenesis. Additionally, Caspase-1 knockdown significantly attenuated atherosclerotic plaque progression in ApoE^−/−^ mice. Overall, our results suggest that macrophage pyroptosis plays an important role in Hcy-induced AS, and inhibition of macrophage pyroptosis may be a potential intervention for Hcy-related AS.

Induction of ERS by Hcy has attracted considerable attention. ERS is a condition in which misfolded proteins accumulate in the ER lumen and is termed the unfolded protein response (Bhardwaj et al. 2020). In mammalian cells, the unfolded protein response is a signaling network consisting of three ER-resident sensors: kinase and endoribonuclease (IRE1), PERK, and the basic leucine zipper activating transcription factor 6 (ATF6) (Di Conza et al. 2020). When exposed to Hcy, macrophages are subjected to ERS and ER-resident kinase and PERK is activated to suppress protein synthesis. Phosphorylation of eIF2α is initiated by PERK and regulates eIF2 complex activity, leading to the expression of transcription factors, such as ATF4, ATF3, and CHOP. In our experiment, we also found that Hcy induced ERS. Furthermore, the ERS inhibitor, 4PBA, attenuated macrophage pyroptosis induced by Hcy. However, there is still a lack of evidence regarding the mechanism by which Hcy promotes pyroptosis. Han et al. demonstrated that CHOP is a potential transcriptional factor that is necessary for NLRP3 expression downstream of PERK. The likelihood that CHOP transcriptionally regulated NLRP3 was strengthened by the chromatin immunoprecipitation assay results, where putative CHOP DNA-binding sites were identified in the promoter region of the NLRP3 gene (Han et al. 2018). Thus, we hypothesize that Hcy may promote macrophage pyroptosis by binding CHOP and the NLRP3 promoter. These results indicate that the PERK pathway contributes to the activation of the NLRP3 inflammasome by ERS through the CHOP-dependent induction of NLRP3. Therefore, we assume that the identified PERK/EIF2α/CHOP pathway is critical for NLRP3 inflammasome-mediated macrophage pyroptosis during AS.

MAM, the functional site between the ER and mitochondria, is involved in lipid metabolism, calcium signaling, mitochondrial fission and fusion, ERS, apoptosis, and autophagy (Gordaliza-Alaguero et al. 2019). Verfaillie et al. reported that without PERK, endogenous apoptosis induced by ERS was weakened due to the reduction in MAM formation and hampered ROS signal transmission to adjacent mitochondria (Verfaillie et al. 2012). This suggested that ERS and MAM could interact with each other, and that the latter was a site for ROS generation. In addition, considerable evidence suggested that in the resting state, NLRP3 was localized in the cytoplasm and ER (Wang et al. 2020). Upon stimulation, the NLRP3 inflammasome was recruited to the MAM sites accompanied by an ASC adaptor. Both ROS generation and NLRP3 inflammasome activation were suppressed when mitochondrial activity was dysregulated by VDAC inhibition, suggesting that MAM played a critical role in NLRP3 inflammasome activation (Zhou et al. 2011). Consistent with previous research, in our study we found that calcium channel-related proteins located on MAMs were enriched and the distance between the ER and mitochondria became closer after Hcy treatment. This indicated that MAM formation increased, and in turn led to elevated ROS production and mitochondrial dysfunction. Therefore, we speculate that Hcy promotes macrophage pyroptosis by tightening the MAM and subsequently causing Ca^2+^ disorder. Our results provide insights into the role of MAM in pyroptosis.

Several models for NLRP3 inflammasome activation have been proposed, such as K^+^ efflux (Kahlenberg and Dubyak 2004), generation of ROS (Zhou et al. 2010), lysosomal destabilization (Hornung et al. 2008), and Ca^2+^ overload (Swanson et al. 2019). However, the precise molecular mechanism of NLRP3 inflammasome activation remains to be elucidated. Studies have shown that calcium-sensing receptor (CASR) activates the NLRP3 inflammasome and is mediated by increased intracellular Ca^2+^ and decreased cellular cyclic AMP. CASR activates the NLRP3 inflammasome through phospholipase C, catalyzing IP3R production, and thereby inducing the release of Ca^2+^ from ER stores (Lee et al. 2012). Increased cytoplasmic Ca^2+^ levels promote the assembly of inflammasome components, which is consistent with our findings. Through MAM, Ca^2+^ is transferred directly from the ER to mitochondria and controls key mitochondrial functions. Indeed, we found that Hcy promoted intracellular and mitochondrial Ca^2+^ overload and led to the rapid collapse of membrane potential and mitochondrial dysfunction. Furthermore, pretreatment with the calcium chelator, BAPTA, protected macrophages from Hcy-induced pyroptosis. Thus, we speculate that Ca^2+^ disorder plays a crucial role in NLRP3 inflammasome activation and macrophage pyroptosis. Furthermore, we found that IP3R-mediated release of Ca^2+^ from endoplasmic reticulum may be the mechanism of Ca^2+^ homeostasis and pyroptosis induced by Hcy. Our results are consistent with previous research (Espitia-Corredor et al. 2022), (Su et al. 2021). Moreover, Other studies have found that there are other ways to regulate calcium overload to activate NLRP3 inflammasome (Nieto-Torres et al. 2015), (Triantafilou et al. 2013), which needs more data to confirm.

In addition, there is considerable evidence that oxidative stress is a potential mechanism that triggers NLRP3 inflammasome activation during pyroptosis (Wu et al. 2020). Excessive ROS concentrations are sensed by NLRP3 and promote the formation and activation of the NLRP3 inflammasome. In our study, Hcy enhanced ER–mitochondrial communication, leading to a massive increase in Ca^2+^ influx into mitochondria. Mitochondrial dysfunction has thus been attributed to Ca^2+^ overload. Hcy also elevated the production of mitochondrial ROS, which was vital for NLRP3 inflammasome activation, inflammatory response, and subsequent pyroptosis. Consistently, we found that NAC inhibited Hcy-induced activation of the NLRP3 inflammasome. Therefore, we conclude that ROS is the critical mechanism that activates the NLRP3 inflammasome.

The present study has some limitations that should be mentioned. First, the current conclusions are all based on in vitro experiments and ApoE^−/−^ mice, and lack of evidence of clinical patient pathology. The level of macrophage pyroptosis in atherosclerotic plaques in HHcy patients should be further analyzed. Second, we focused on the role and potential mechanism of Hcy inducing pyroptosis of macrophages. In our study, only THP-1-derived macrophages were used, and murine macrophages were not used for further verification. Third, to exclude the protective effect of estrogen on atherosclerosis, only male ApoE^−/−^ mice were used to construct the atherosclerosis model in the study. Therefore, our current conclusions may be restrictive for female mice.

**Fig. 8:**
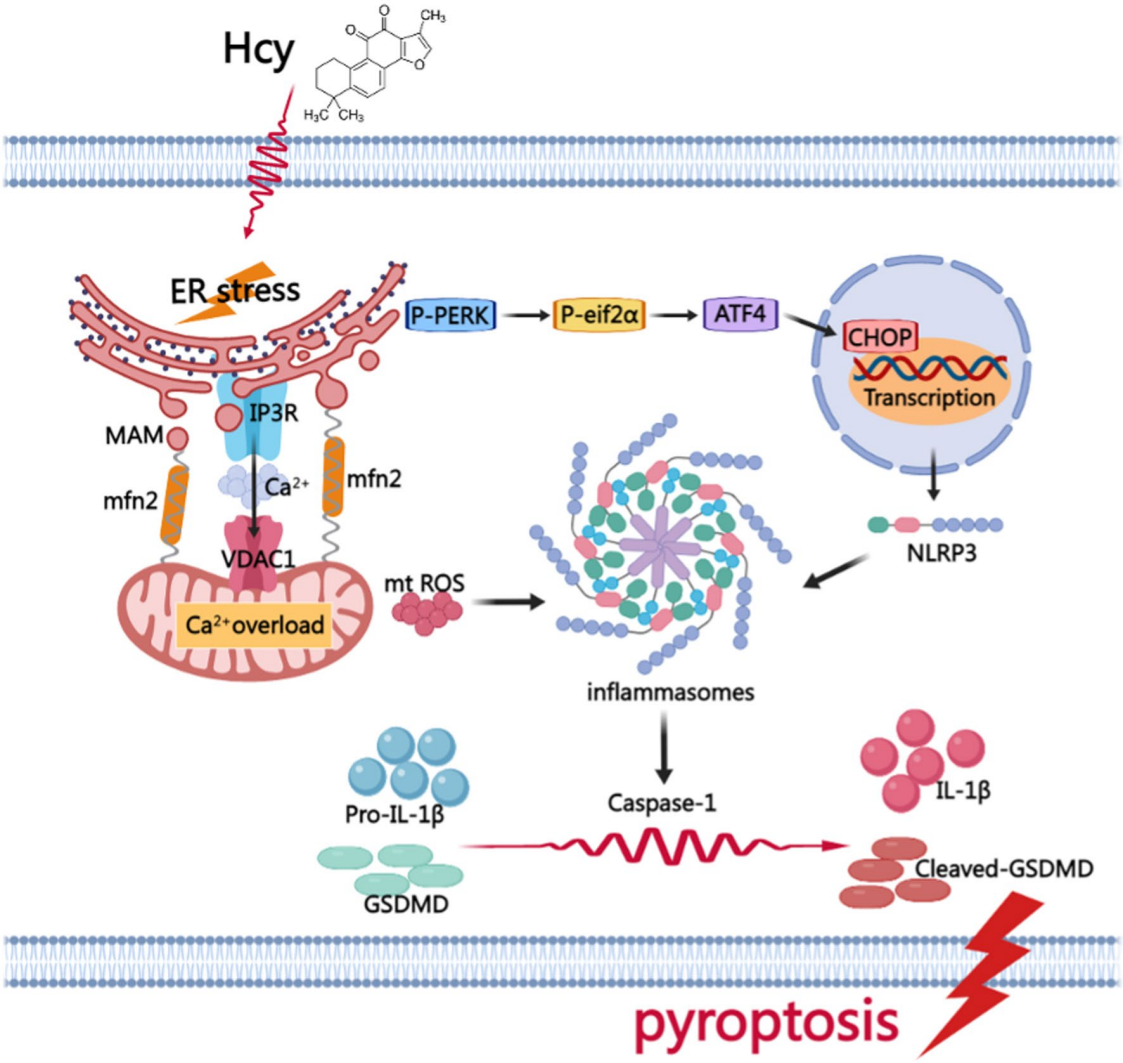
A model hypothesis diagram demonstrates the possible mechanism of Hcy promoting macrophage pyroptosis to accelerate atherosclerosis

Taken together, our findings are the first to report that Hcy induces macrophage pyroptosis via ERS and Ca^2+^ disorder, thus promoting AS progression. Hence, pyroptosis is likely a cellular mechanism underlying the detrimental effects of Hcy on AS, with the production of ROS and activation of NLRP3 as upstream mediators. Additionally, our study is the first to demonstrate that Hcy induces ERS activation and enhances ER-mitochondrial coupling, which provides a channel for Ca^2+^ massive transfer to the mitochondria. We demonstrate that Ca^2+^ overload causes mitochondrial dysfunction and stimulates ROS generation, and which is vital for NLRP3 inflammasome activation. Moreover, we know that Ca^2+^ can directly activate NLRP3 inflammasome through CASR. In addition, ERS activates the NLRP3 inflammasome via the immediate interaction between CHOP and NLRP3 (Fig. 8). Targeting Caspase-1-dependent pyroptosis may be a new approach for alleviating atherosclerotic lesions induced by Hcy. This work provides supporting evidence for the important role of organelle interaction in regulating macrophage death and sheds new light on the mechanisms underlying the pathogenesis of HHcy-accelerated AS.

## Conclusions

Taken together, the present results demonstrated that Hcy accelerates atherosclerosis progression by enhancing macrophages pyroptosis via promoting endoplasmic reticulum stress, endoplasmic reticulum-mitochondria coupling, and disturbing of calcium disorder. Our study provides new insights into the molecular mechanisms of Hcy in promoting atherosclerosis.

## Supplementary Information

**Additional file 1: Fig. S1.** The basic effects of methionine in ApoE-/- mice.Body weight in ApoE-/- mice with different diets as indicated. Food intake in three different groups. Blood glucose in three different groups. Plasma TC content in three different groups. Plasma TG content in three different groups. Plasma LDL content in three different groups. Plasma HDL content in three different groups. The data are shown as the mean±SD. *P<0.05, **P<0.01. **Fig. S2.** IP3R inhibitor represses Hcy-induced macrophage pyroptosis. The cell death was measured with Hoechst 33342/PIdouble-fluorescent staining. The scale bars correspond to 100 μm.The morphology of cells was observed with scanning electron microscopy. The scale bars correspond to 10 μm.Cell viability detected by CCK8.LDH assay was used to evaluate the cell membrane integrity.Ca2+ change with the fluorescence. The levels of cellular and mitochondrial calcium were measured by Fluo-4 and Rhod-2 respectively. The scale bars correspond to 100 μm.Western blot was used to evaluate the pyroptosis-related proteins level in different groups. The data are shown as the mean±SD. *P<0.05, **P<0.01.

## Data Availability

The data in the current study are available from the corresponding author on reasonable request.

## Abbreviations

AS: Atherosclerosis
ATP: Adenosine 5′-triphosphate
CASR: Calcium-sensing receptor
ECs: Endothelial cells
ERS: Endoplasmic reticulum stress
Hcy: Plasma homocysteine
HFD: High-fat diet
HHcy: Hyperhomocysteinemia
HM: High-fat plus methionine
IP3R: Inositol-3-phosphate receptor
LDH: Lactate dehydrogenase
MAM: Mitochondria-associated endoplasmic reticulum membrane
MMP: Mitochondrial membrane potential
NAC: N-acetyl cysteine
PERK: Protein kinase R-like endoplasmic reticulum kinase
PI: Propidium iodide
ROS: Reactive oxygen species
TC: Total cholesterol
TG: Triglyceride
VDAC: Voltage-dependent anion channel
