# Supplementary material for: Homocysteine promotes atherosclerosis through macrophage pyroptosis via endoplasmic reticulum stress and calcium disorder
Source: Mol Med. 2023 Jun 12;29:73. doi: 10.1186/s10020-023-00656-z (PMC10262416; doi:10.1186/s10020-023-00656-z)
Supplement: Supplementary file 1 — Additional file 1: Fig. S1. The basic effects of methionine in ApoE-/- mice.Body weight in ApoE-/- mice with different diets as indicated. Food intake in three different groups. Blood glucose in three different groups. Plasma TC content in three different groups. Plasma TG content in three different groups. Plasma LDL content in three different groups. Plasma HDL content in three different groups. The data are shown as the mean±SD. *P<0.05, **P<0.01. Fig. S2. IP3R inhibitor represses Hcy-induced macrophage pyroptosis. The cell death was measured with Hoechst 33342/PIdouble-fluorescent staining. The scale bars correspond to 100 μm.The morphology of cells was observed with scanning electron microscopy. The scale bars correspond to 10 μm.Cell viability detected by CCK8.LDH assay was used to evaluate the cell membrane integrity.Ca2+ change with the fluorescence. The levels of cellular and mitochondrial calcium were measured by Fluo-4 and Rhod-2 respectively. The scale bars correspond to 100 μm.Western blot was used to evaluate the pyroptosis-related proteins level in different groups. The data are shown as the mean±SD. *P<0.05, **P<0.01. [file 10020_2023_656_MOESM1_ESM.docx]

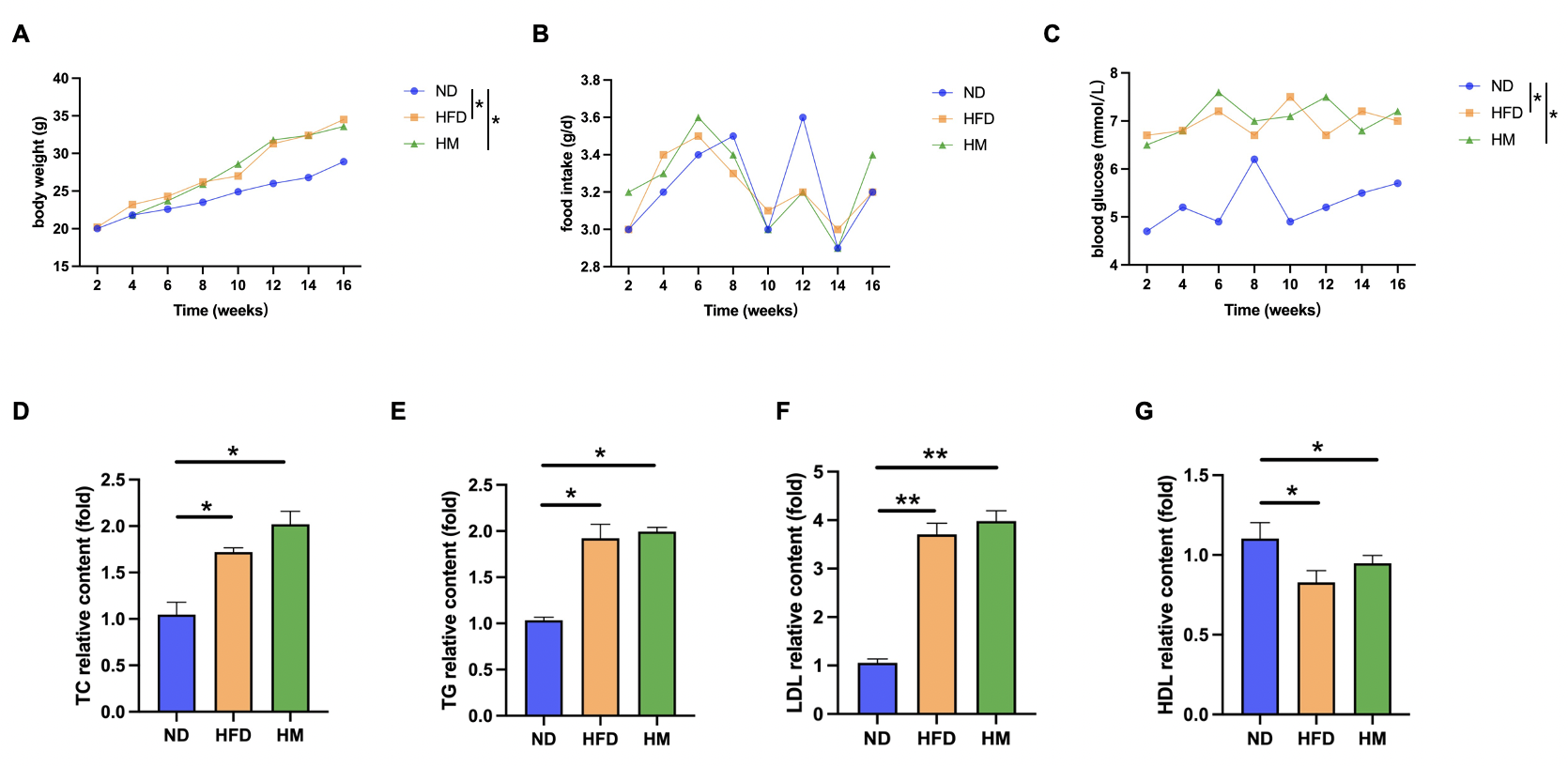


**Additional file 1.**

**Fig. S1 The basic effects of methionine in ApoE^-/-^ mice.**

(A) Body weight in ApoE^-/-^ mice with different diets as indicated (n=10). (B) Food intake in three different groups (n=10). (C) Blood glucose in three different groups (n=10). (D) Plasma TC content in three different groups (n=10). (E) Plasma TG content in three different groups (n=10). (F) Plasma LDL content in three different groups (n=10). (G) Plasma HDL content in three different groups (n=10). The data are shown as the mean±SD. *P<0.05, **P<0.01.

**
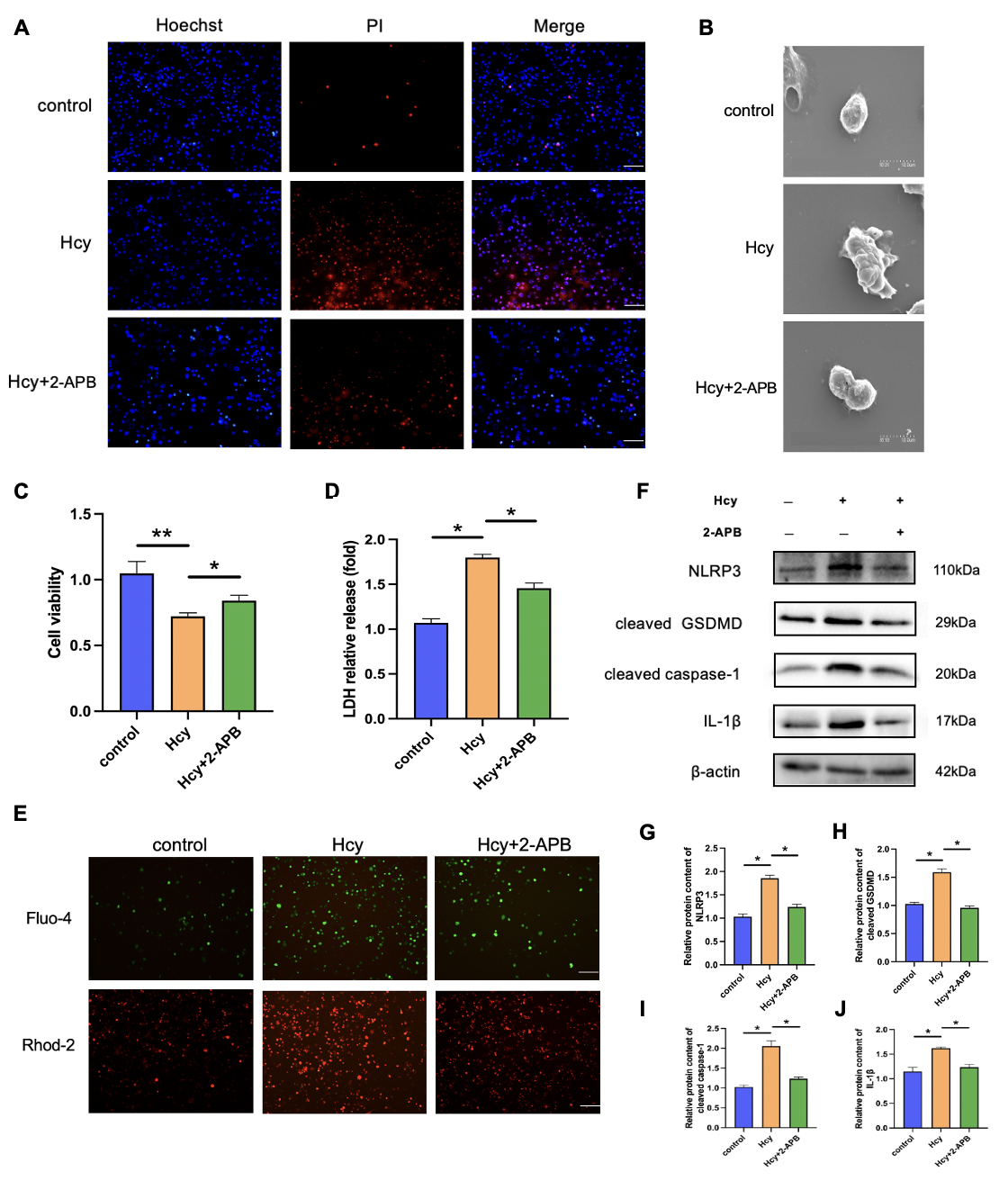
**

**Fig. S2. IP3R inhibitor** **represses Hcy-induced macrophage pyroptosis.**

(A) The cell death was measured with Hoechst 33342 (blue)/PI (red) double-fluorescent staining. The scale bars correspond to 100 μm. (B) The morphology of cells was observed with scanning electron microscopy (SEM). The scale bars correspond to 10 μm. (C) Cell viability detected by CCK8. (D) LDH assay was used to evaluate the cell membrane integrity. (E) Ca^2+^ change with the fluorescence. The levels of cellular and mitochondrial calcium were measured by Fluo-4 and Rhod-2 respectively. The scale bars correspond to 100 μm. (F-J) Western blot was used to evaluate the pyroptosis-related proteins level in different groups. The data are shown as the mean±SD. *P<0.05, **P<0.01.
